# Cervicovagopathy: ligamentous cervical instability and dysstructure as a potential etiology for vagus nerve dysfunction in the cause of human symptoms and diseases

**DOI:** 10.3389/fneur.2025.1572863

**Published:** 2025-07-02

**Authors:** Ross A. Hauser, Danielle Matias, Benjamin Ryan Rawlings

**Affiliations:** Caring Medical Florida, Fort Myers, FL, United States

**Keywords:** vagus nerve dysfunction, vagus nerve injury, cervicovagopathy, ligamentous cervical instability, dysautonomia, heart rate variability, prolotherapy

## Abstract

Vagus nerve dysfunction is implicated in the pathophysiology of many different symptoms and diseases that plague humanity. In many cases, the etiology of this condition remains elusive. One potentially implicating factor is cervical spine pathology, as the 2 vagus nerves are located in the carotid sheath just anterior to the cervical vertebrae. We propose that cervicovagopathy occurs primarily by the slow stretching of the posterior cervical ligaments because of a forward head-facedown lifestyle from excessive cell phone and computer usage. While the excessive stretch and compression on the vagus nerve initially just inhibits electrical impulses (conduction block), the condition progresses to ligamentous cervical instability. It ultimately results in a breakdown of the cervical curve (dysstructure), leading to vagus neuron cell death (degeneration), which can be documented by carotid sheath ultrasound. Cervical structural, internal jugular vein, and vagus nerve cross-sectional area measurements are presented from a retrospective chart review of 234 consecutive patients with no obvious cause for 1 of 9 specific symptoms—anxiety, dizziness, fatigue, irritability, lightheadedness, insomnia, sleeping difficulty, neck pain, and neck cracking/popping. Those cases of vagus nerve degeneration from a structural cause require corrective cervical structural therapies such as proper ergonomics, physiotherapy, cervical curve and postural exercises, low-force adjustments, and prolotherapy. A case example is given to demonstrate how cervical structural treatments can open up internal jugular veins and improve a patient’s chronic symptoms. Resolution of symptoms that occur alongside improvements in vagus nerve cross-sectional areas (regeneration), correlating with restoration of the cervical lordotic curve and stability, will prove this hypothesis.

## Introduction

Operating far below the level of our conscious minds, the vagus nerve (VN) is vital for keeping our bodies healthy. The VN is our sixth sense, our “gut feeling” (1). It keeps us alive by its innervation of the internal organs of the body and their interactions with the brain, spinal cord, cranial nerves, upper cervical spinal nerves, and sympathetic nervous system. The VN innervates all the portals of entry or filters for pathology from toxins, microorganism invaders, and allergens that can enter the human body, namely the respiratory and gastrointestinal epithelial mucosal surfaces. Since the VN meanders and extends branches to multiple tissues and organs, its role in health is immense, regulating homeostasis by connecting 3 interwoven systems—the nervous, endocrine, and immune systems.

**Figure 1:**
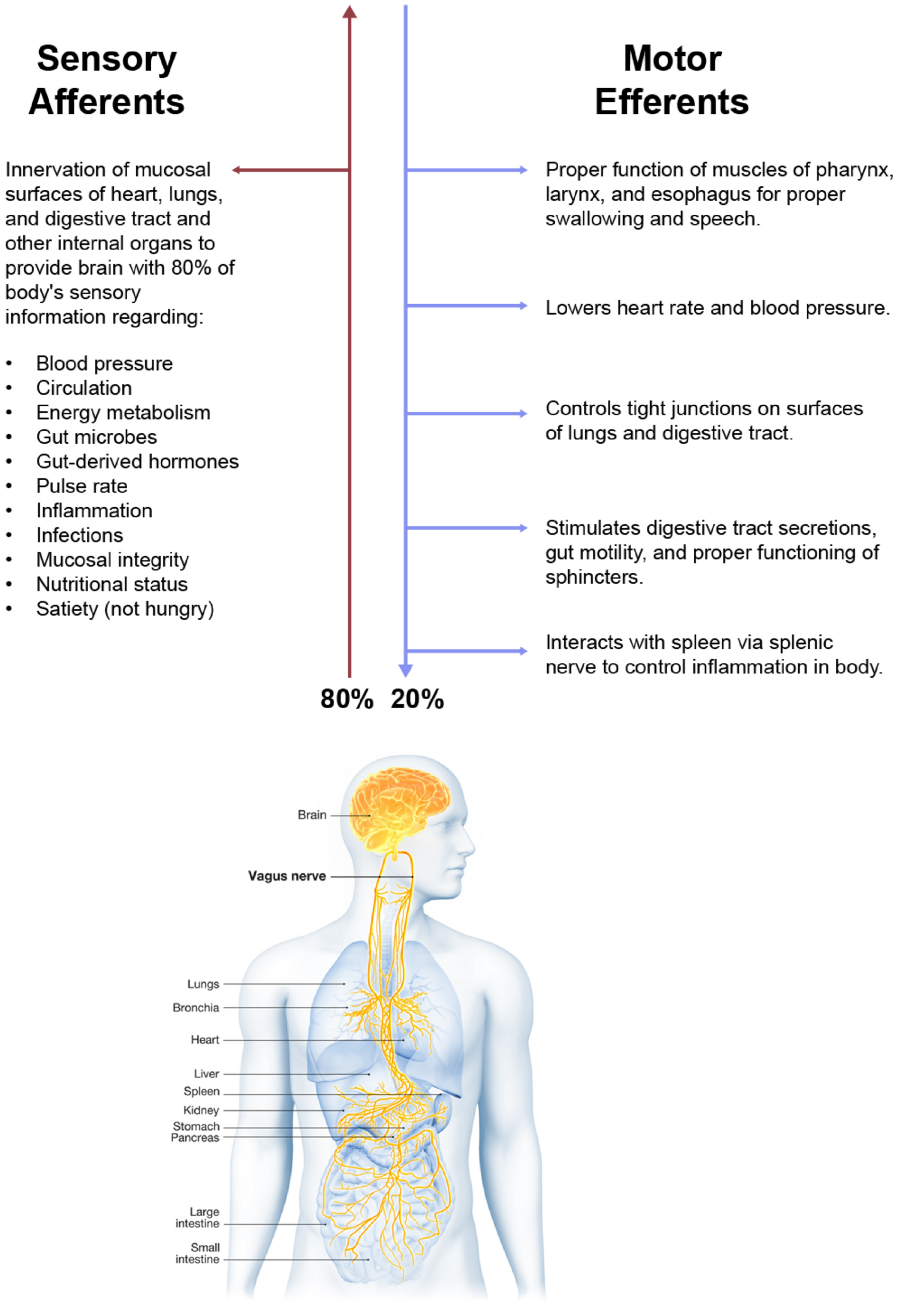
The vagus nerve.

In Latin, *vagus* means a “fugitive” or “wanderer.” It is the longest and most widely extended of the nerves of the body, carrying both sensory and motor information to and from the brain, traversing through the neck to innervate the organs (see Figure 1). The VN works as a 2-way messenger, passing electrochemical signals between the organs and brain regarding heart rate, blood pressure, circulation, breathing, internal organ distension, secretions, and inflammation. In the neck, the VN has direct connections to the inferior, middle, and superior cervical sympathetic ganglia, as well as the upper cervical nerves and many of the cranial nerves and descends all the way down to the celiac plexus in the abdomen and beyond (2).

**Figure 2:**
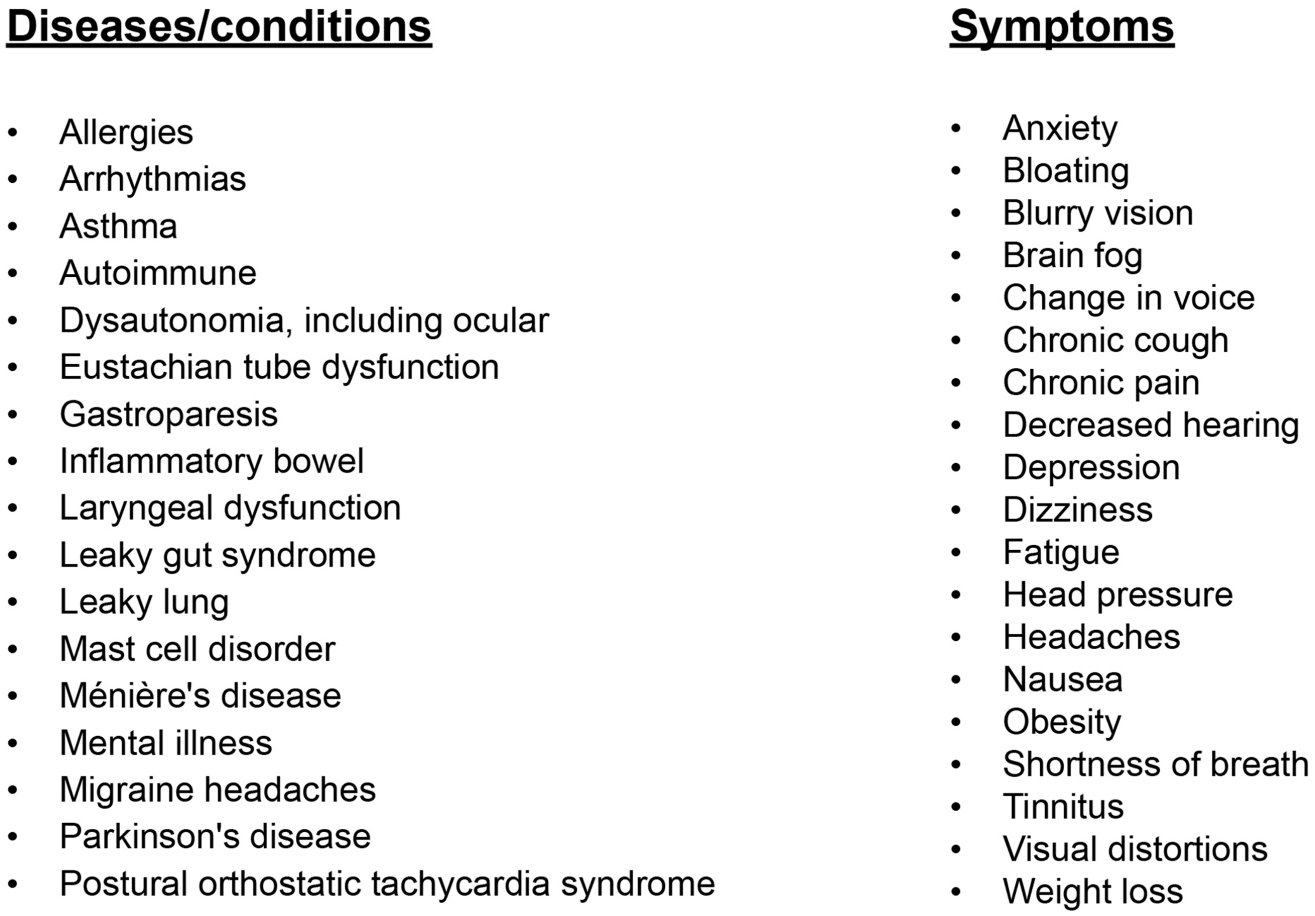
A list of diseases/conditions/symptoms that potentially involve the vagus nerves.

The role of vagus nerve function in health and disease cannot be overemphasized. The hallmarks of dysautonomia (parasympathetic dysfunction causing sympathetic dominance, or what is termed “sympathovagal imbalance”) are paramount to understanding the pathophysiology of most medical conditions (3, 4). The cause of vagal or parasympathetic dysfunction has many chemical and emotional etiologies, including diabetes, heavy metals, medications, and emotional or financial stress, but a cause that is overlooked is structural injury (5). As the list of symptoms and diseases continues to grow, including chronic pain, depression, tinnitus, migraine headache, seizures, heart failure, Alzheimer’s dementia, and systemic inflammation, it is prudent that clinicians and the patients they treat understand the VN anatomy, injury, and the potential pathophysiology it causes (6–8) (see Figure 2).

## Pertinent vagus nerve cervical anatomy

The right and left vagus nerves are most vulnerable to stretch, traction, and compression in the cervical region, as they lie in a specific area called the carotid space. The carotid space is a paired space defined by the carotid sheath, a connective tissue boundary in the neck that is made up of superficial, middle, and deep layers of the cervical fascia (9). The suprahyoid (above hyoid bone) area of the carotid space contains the internal carotid artery, internal jugular vein (IJV), cranial nerves IX-XII, the ansa cervicalis (a loop of the first 3 cervical nerves), the sympathetic plexus, and deep cervical lymph nodes (10).

**Figure 3:**
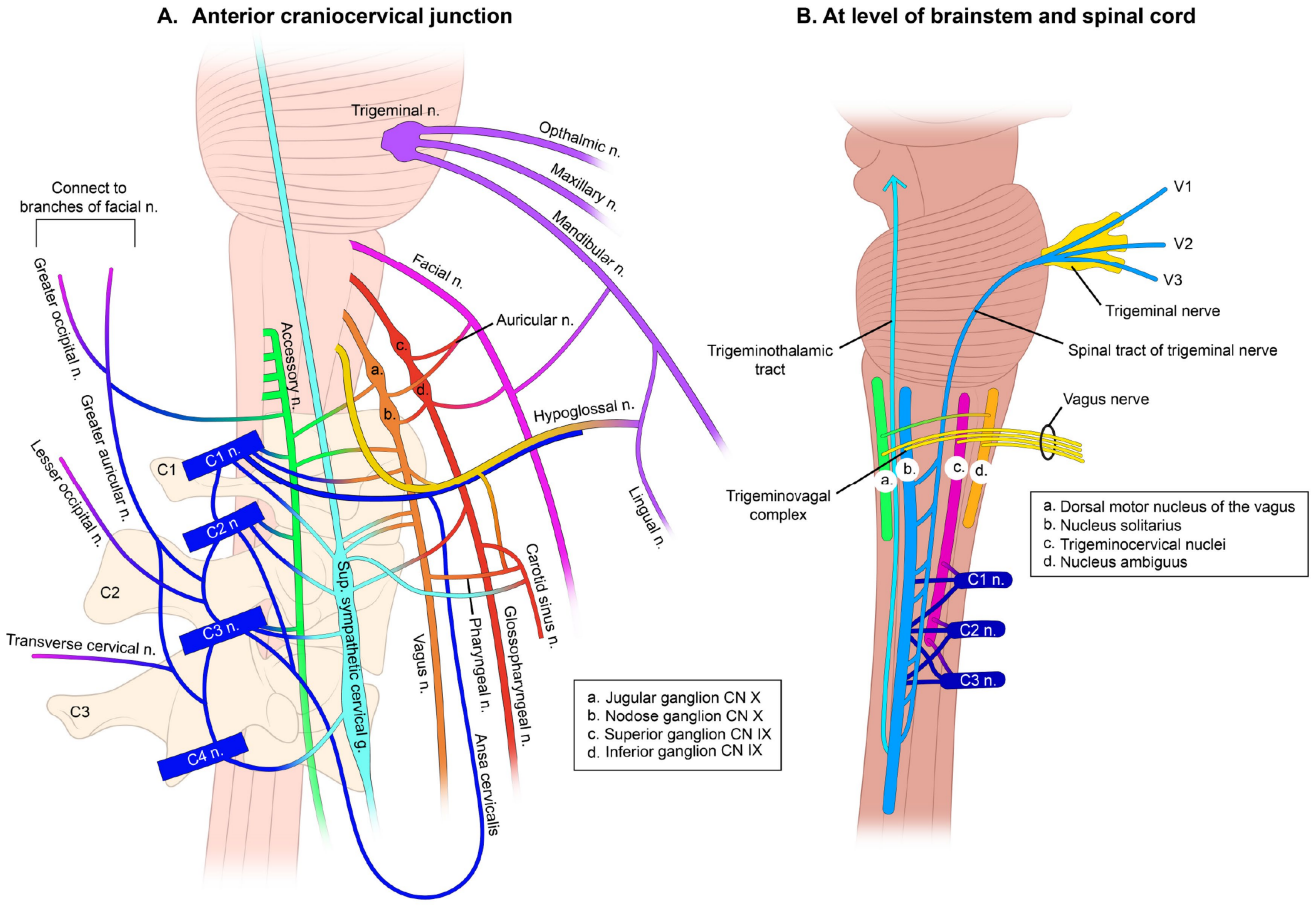
Interconnectedness of vagus nerves. **(A)** Anterior craniocervical junction (a. Jugular ganglion CN X, b. Nodose ganglion CN X, c. Superior ganglion CN IX, d. Inferior ganglion CN IX). **(B)** At level of brainstem and spinal cord (a. Dorsal motor nucleus of the vagus, b. Nucleus solitarius, c. Trigeminal nuclei, d. Nucleus ambiguus).

The peripheral hub of the whole autonomic nervous system is the upper cervical spine, as the inferior (nodose) ganglion of the VN lies right in front of C1, and the superior cervical sympathetic ganglion (SCSG) lies just anterior to C2 and C3 (11, 12). In the upper cervical region, the vagus neurons connect with the trigeminal, facial, glossopharyngeal, spinal accessory, and hypoglossal nerves (cranial nerves V, VII, IX, XI, and XII, respectively), along with the connections to the cervical sympathetic trunks and C1–C3 spinal nerve roots (13, 14). One especially important aspect is the inhibitory effect of the VN on the SCSG (see Figure 3). The SCSG innervates the eye and lacrimal gland, causes vasoconstriction of the iris and sclera, pupillary dilation, and widening of the palpebral fissure, and reduces tear production. It has been implicated in many conditions and symptoms that include elevated intraocular pressure, glaucoma, photophobia, and macular degeneration (15–17).

**Figure 4:**
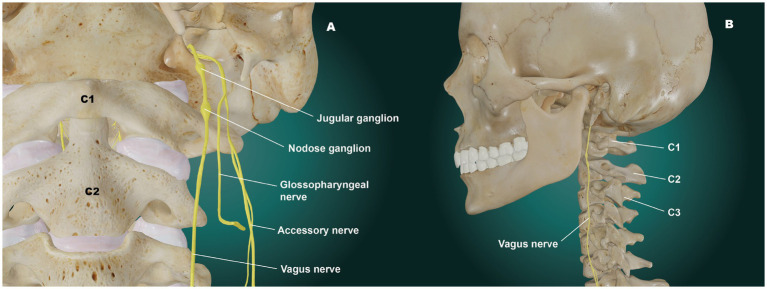
The cervical vagus nerves. **(A)** Anterior view. **(B)** Lateral view. The vagus nerves run dangerously close to the anterior cervical vertebrae, which is especially true at the atlanto-axial joint, where it is very vulnerable to traction stretch.

The nodose ganglion provides vital sensory information such as mechanoreception (stretch) and nociception (pain) from the ear, tympanic membrane, and parts of the dura mater that interact with the spinal trigeminal nucleus of the brainstem, its importance cannot be overemphasized. It surveys the physiological state of the internal body by relaying sensory information from the larynx, heart, lungs, and gastrointestinal tract to the brainstem and brain (18). The nodose is approximately 5 times the size of the jugular ganglion, is the key sensor of the parasympathetic nervous system of the body and is the ganglion that sits right in front of C1 (19) (see Figure 4). These neurons in the nodose ganglion are critical in relaying information such as elevations in blood pressure, changes in blood oxygenation and respiratory rate, passage of contents through the esophagus and intestines, and distention of the heart, stomach, and lungs to the dorsal nuclei of the VN in the medulla and central nervous system for optimization of visceral function health. Each nodose ganglion neuron in the digestive tract interacts with thousands of enteric neurons for coordination of optimum digestion, absorption, appetite, and systemic changes in energy utilization. These neuronal pathways influence the release of hundreds of metabolic hormones and neurotransmitters, blood glucose levels, enzyme secretion, gallbladder contraction, gut motility, gastric acidification, gastric emptying, hydration status, and nutrient levels, as well as assessing microbiome-derived metabolites and other potential pathogens, toxins, or food allergens (18, 20, 21).

Fully 80% of parasympathetic sensory afferents are from the VN (cranial nerve X), a mixed nerve composed of 20% efferent fibers sending signals from the brain to the body and 80% afferent (sensory) fibers carrying information from the body to the brain (22, 23). A key point is that because the VN is the body’s sensor, it is primarily through the VN that the brain knows what is happening in and to the body.

Vagus neurons need to be kept healthy and alive, as there are not as many of them compared to other tissues. The VN is extremely small, given its massive importance. In 1961, Drs. Hoffman and Schnitzlein published that the number of nerve fibers in the mid-cervical vagus nerve of man varied from 45,110 to 153,123 fibers (right average was 105,375, left average was 87,300) (24). The VN has 1/1,000th of the number of nerve fibers as the enteric nervous system. There are approximately 1 million neurons in each eye, 100 + million in the spinal cord, and 100–500 million neurons in the enteric nervous system, 0.1% the number of neurons in the brain (100 billion) (25, 26).

## Forward head-facedown lifestyle demise of the cervical spine structure

An estimated 129 million people in the United States—over 35% of the population—have at least one major chronic disease (27). The prevalence of chronic disease in the United States has steadily increased over the past 2 decades, with 42% of people having 2 or more chronic diseases, and 12% having at least 5 diseases (28). While there are many contributing factors, including obesity and stress, what are often overlooked are dysfunctional changes in the cervical curve that occur with a forward head-facedown lifestyle due to excessive screen time on electronic devices.

**Figure 5:**
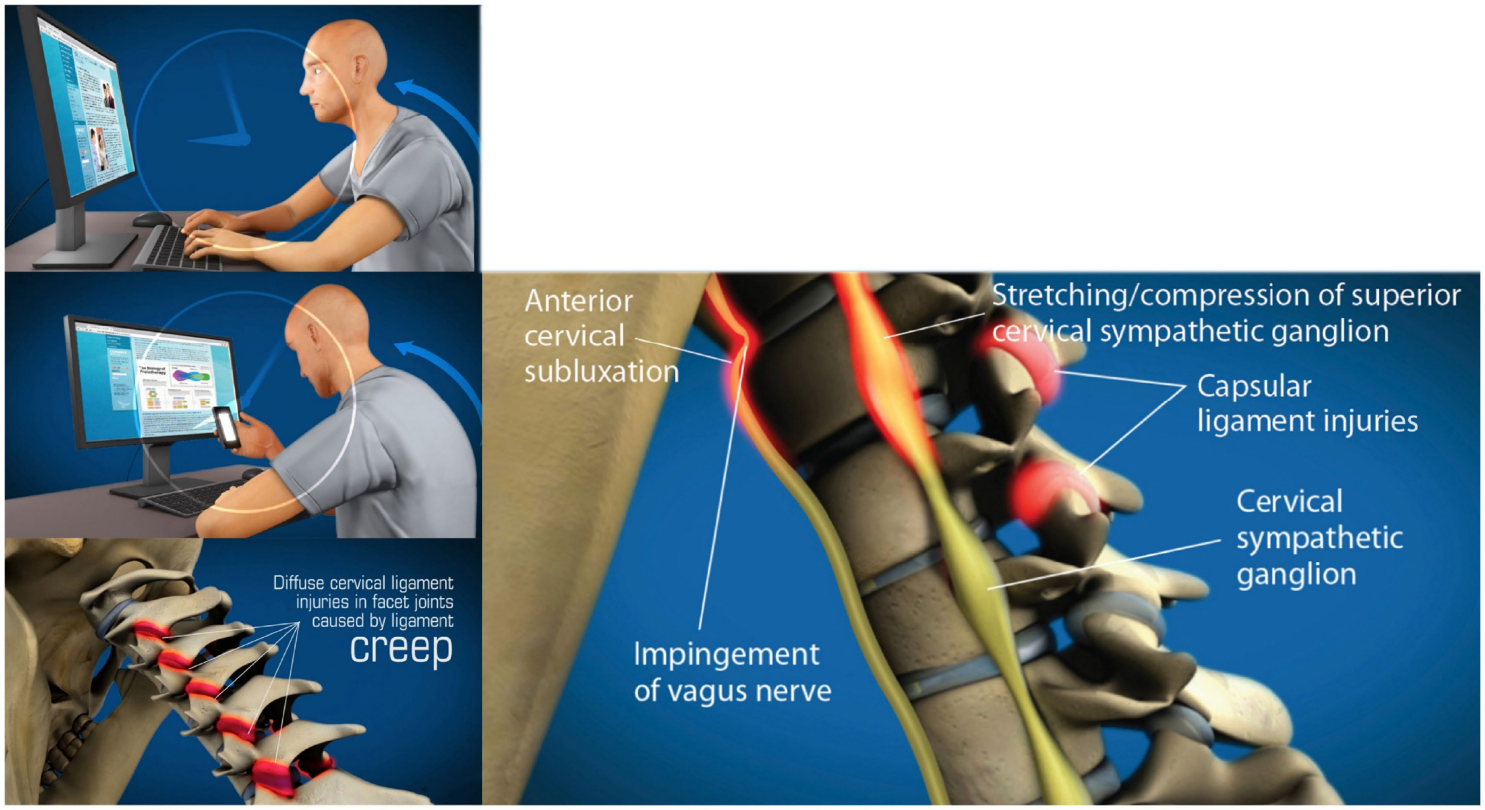
Forward head posture from hours of computer work and texting, resulting in cervical ligament laxity, ultimately compressing the internal jugular veins and vagus nerve. “Creep,” which is a term signifying the slow stretching of ligaments, most commonly occurs by a forward head posture from computer work or looking at a smartphone. As cervical vertebrae sublux anteriorly, a stretch compression can occur on the internal jugular veins and autonomic nerves in the anterior part of the neck, including the vagus nerves and cervical sympathetic ganglion.

The forecast for the number of mobile cell phone users worldwide is expected to be approximately 7.5 billion in 2025 (29). Daily time spent in front of electronic devices continues to rise, up 60% since 2020, as Americans currently spend almost 7 h per day online, and 92% of jobs in the U. S. require digital skills (30). This prolonged forward head-facedown lifestyle alters spinal posture, especially in the cervical spine, because of slow stretching of the posterior ligament complex of the neck, a process known as “creep” (31) (see Figure 5).

**Figure 6:**
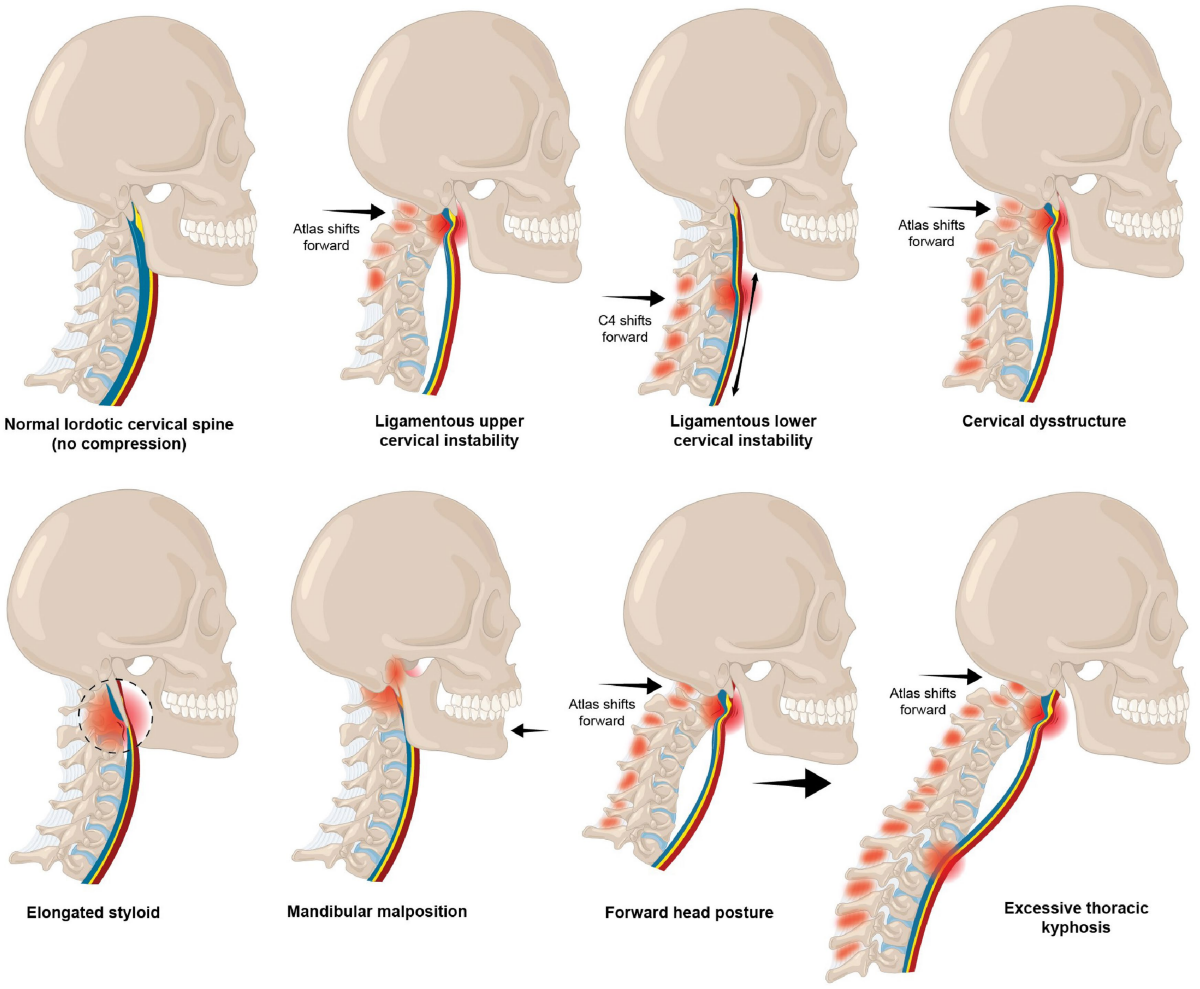
Various types of structural dynamic internal jugular vein and vagus nerve compression and stretch.

Inappropriate poor neck posture while looking at a computer screen or texting leads to the manifestation of a host of musculoskeletal, mental health, emotional, and body symptoms commonly known as “text neck syndrome” (32). Forward head posture is the most common cervical postural dysfunction and is associated with myriad symptoms and diseases, including cervicogenic dizziness, vertigo, migraines, and even a decrease in brain function (33–35). The average 5–7 h that people, including children and adolescents, spend looking down at their cell phones potentially causes multifarious changes in the cervical spine, including an elongation or stretching of the posterior ligament complex. This stretch causes ligamentous cervical instability and a breakdown and loss of the cervical curve (cervical dysstructure), as well as overall changes in the sagittal plane, where the upper cervical spine is forward in relation to the lower cervical spine: the very definition of forward head posture (36, 37). While this is likely the primary mechanism causing structural vagus nerve dysfunction, it could also occur because of mandibular malposition and elongated styloid bones (see Figure 6).

The cervical region is the spine’s most mobile segment. Its primary stability stems from its ligament structures, especially in the upper cervical region, which is devoid of discs. The forward head posture puts the lower neck (C2–C7) into constant flexion, which necessitates the upper cervical area (C0–C2) to be in extension in order to maintain a stable horizontal gaze (35, 38). The larger the neck flexion angle, the greater the forces on the posterior soft tissue structures in the neck, making cervical instability more plausible (39–42). Ultimately, the upper cervical spine at the atlas subluxes forward, the net effect of these changes resulting in compression of the carotid sheath and its contents at the level of the atlas, including the IJV and VN (43).

## Vagus nerve degeneration and conduction block

Vagus nerve degeneration signifies that the electrical impulses in the nerve are hampered, which implies either conduction block or loss of vagal neurons, termed vagopathy or degeneration. Excessive forces on a nerve, whether by stretch or compression, can initially block nerve impulses, but if not removed, will ultimately cause neuron cell death. As little as 6% stretch of a nerve has been shown to block conduction (44). It is well known that cervical flexion significantly lengthens/stretches the cervical spinal cord and nerve roots (up to 18%) and the same must be assumed for the VN (45–47).

**Figure 7:**
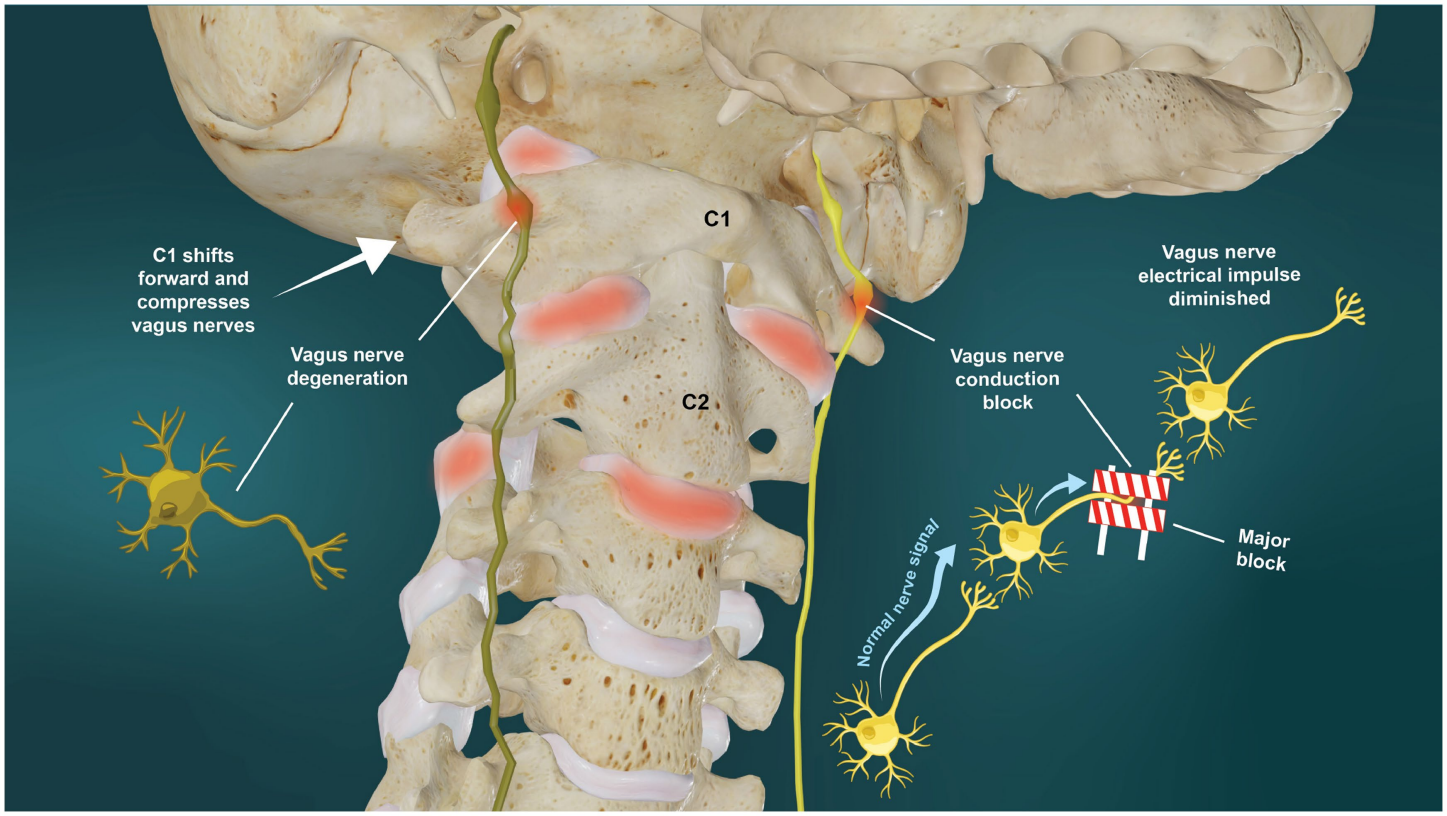
Ligamentous upper cervical instability potential etiology of vagus nerve dysfunction. Vagus nerve dysfunction can result from degeneration or conduction blocks from stretch and compression on the nerve at the level of the atlas (C1).

When a nerve is subjected to compression or stretch forces long-term, whether from changes in bony or muscular anatomy or within the nerve sheath (cerebrospinal fluid) or the nerve itself (arterial/venous compromise, swelling), neuron cell death can occur, with the larger-diameter fibers typically affected first (48, 49). It has been shown that both slow and fast axonal transport are impaired in the cervical VN by low pressures of around 20–30 mmHg, which are comparable with those found in human compression neuropathies, such as carpal tunnel syndrome (50). Compression of the VN at 20, 30, and 200 mmHg can induce a graded inhibition of both retrograde and anterograde transport of radiolabeled proteins (51). In one study, even slight trauma to the nerve, represented by a pressure at 50 mmHg applied for 2 h, induced accumulation of axonally transported proteins at the level of compression (52). This accumulation caused nerve transmission to be blocked for up to 1 day. When the VN compression was applied for 2 h at a pressure of 400 mmHg, the conduction block lasted up to 3 days. Conduction block and/or vagus nerve degeneration would both have the net effect of causing vagus nerve dysfunction (see Figure 7). Stretch or other deformation injuries to the axons can cause loss of microtubules and neurofilaments, loss of axon transport, and the accumulation of toxic substances that can destroy either transport or the axon itself (53). The carotid sheath contents, including the VN, are highly vulnerable to tissue strain by deformations or deviations from the normal, stable cervical lordotic curve due to their location and length, as the vagus nerves run just anterior to the anterior vertebral bodies.

## In-office vagus nerve testing

**Figure 8:**
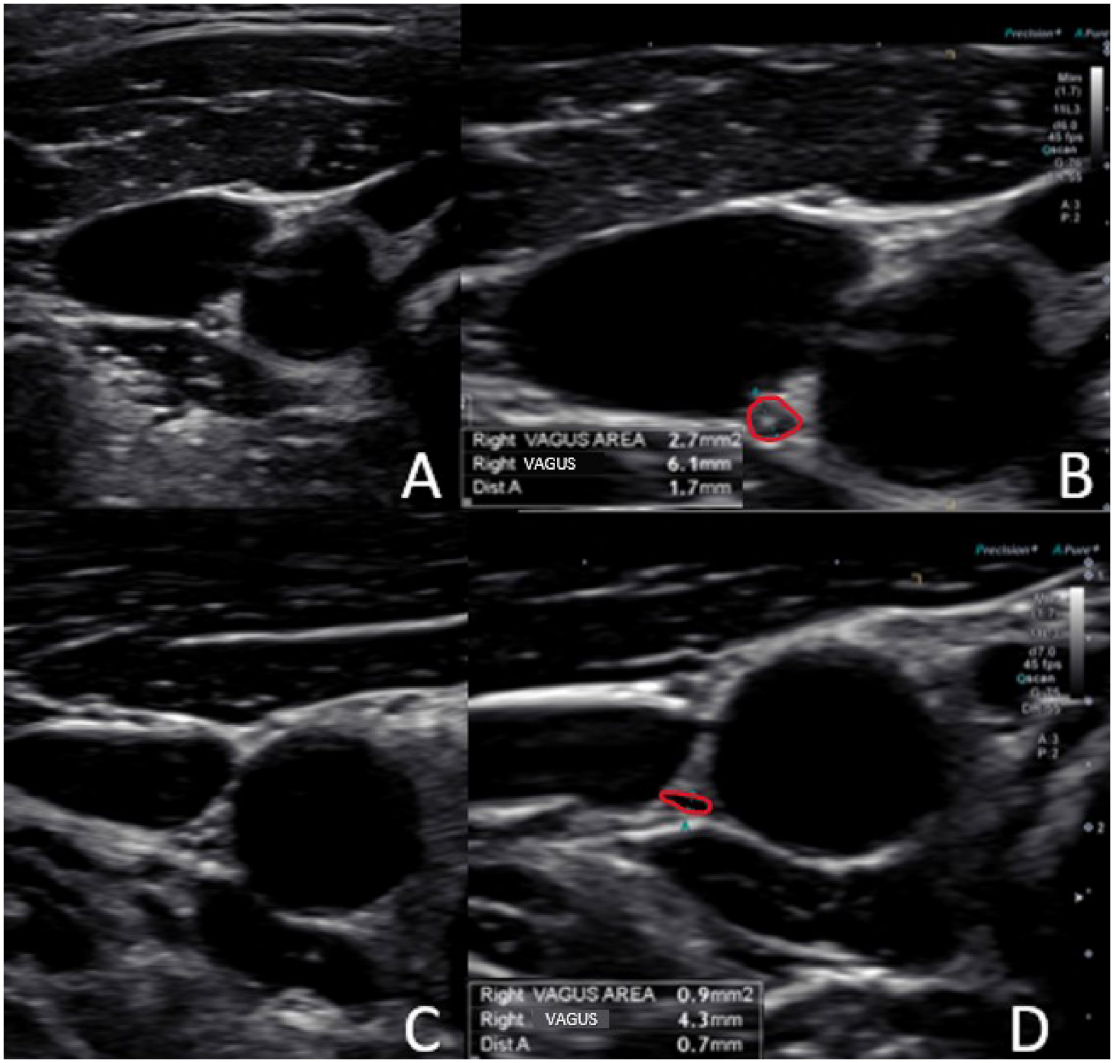
Ultrasound of the carotid sheath showing the vagus nerve. **(A)** Normal. **(B)** Normal image magnified and measured. **(C)** Degenerated vagus nerve. **(D)** Degenerated image magnified and measured.

**Figure 9:**
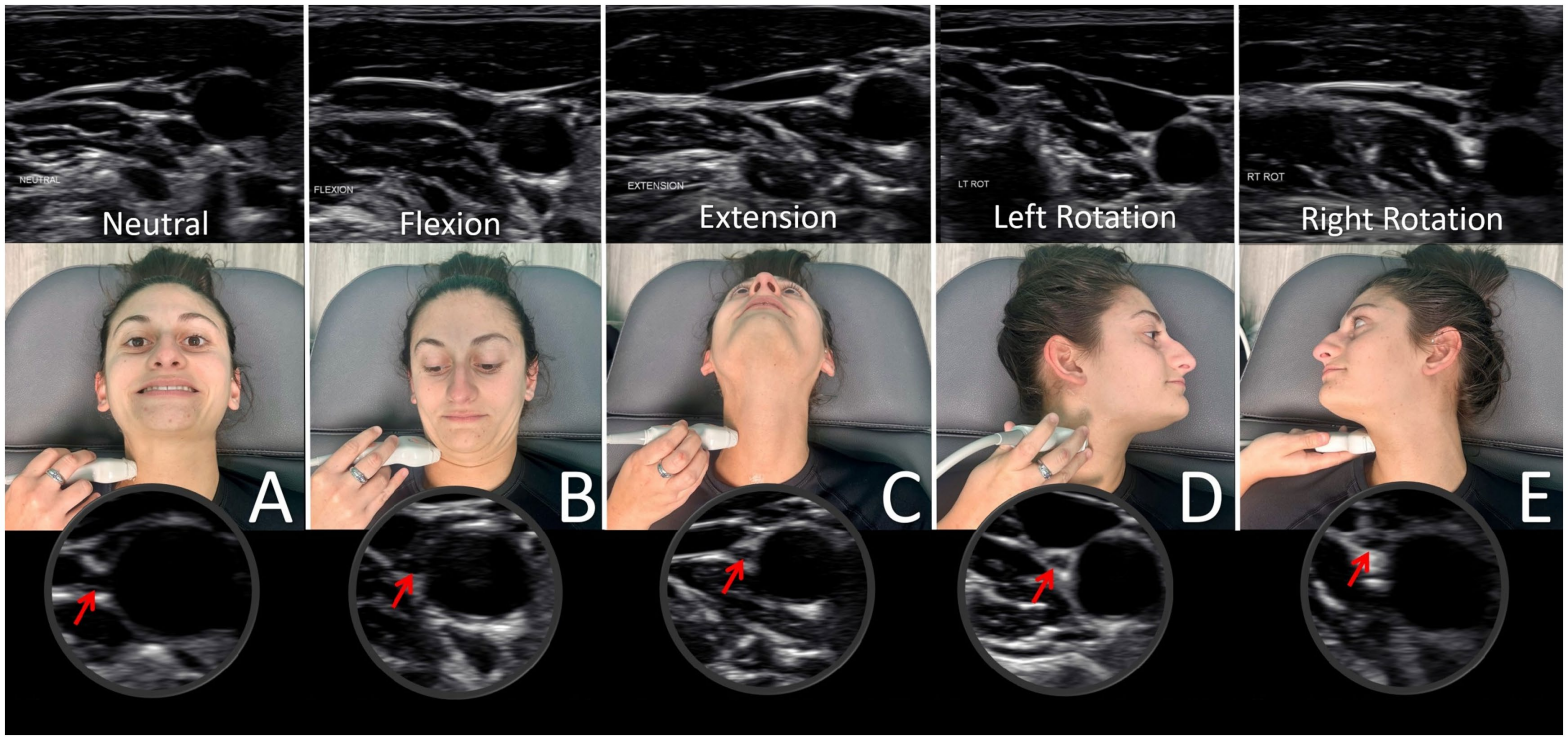
Ultrasound of vagus nerve in mid-cervical region with various neck positions. **(A)** Neck in neutral position. **(B)** Neck flexed. **(C)** Neck extended. **(D)** Neck rotated left. **(E)** Neck rotated right. As can be seen, the vagus nerve within the carotid sheath undergoes various structural tensions depending on neck positions.

Using high-resolution ultrasound, the VN is easily examined, with the most common location of the nerve in the mid-neck lying posterior to the IJV and lateral to the carotid artery (54). The VN cross-sectional area can then be measured (see Figure 8). Normal cross-sectional areas are between 2 and 3 mm (2, 55–58). Studies have shown that the right VN is significantly larger than the left (59). Vagus nerve degeneration is documented by a decrease in the cross-sectional area on ultrasound in the mid-cervical region. The VN cross-sectional area has been shown to decrease with age, as well as with various diseases (up to 30%), including Parkinson’s disease, diabetes, alcoholic-induced dysautonomia, and amyotrophic lateral sclerosis, and can be correlated with symptomatology (60–63). Stretch and tension on the VN can be seen on ultrasound by changes in configuration at various cervical levels and with different head/neck positions (see Figure 9).

## Vagus nerve degeneration in cohort of consecutive patients

Measurements were taken for 232 consecutive patients aged 20–50 (avg. 37.2 yrs., 50.2% male [n = 121]) going to an outpatient neck center from January 1, 2022 to June 30, 2022 with no obvious cause, including previous traumas, for at least 1 of 9 symptoms: anxiety, dizziness, fatigue, irritability, lightheadedness, insomnia, sleep difficulty, neck pain, and neck cracking/popping (see Table 1). This retrospective study was approved by the WCG Institutional Review Board (Study #1364545). The testing process was previously described (64). The cervical instability and dysstructure found are presumed to “simply” be from a forward head-facedown lifestyle with computer and cell phone usage. The small VN cross-sectional area seen from carotid sheath compression at the atlas is due to a combination of forward head posture and ligamentous upper cervical instability.

**Table 1:** Cervical structural analysis and vagus nerve cross-sectional area (CSA) of 232 consecutive patients with various symptoms going to an outpatient neck center.

Vitals								
Symptoms	*N*	Vagus Nerve CSA^a^ (nl > 4.2 mm^2^)	IJV CSA C1, Supine (nl > 180 mm^2^)	Depth of Curve^b^ (nl 7–17 mm)	C6AI^c^ (nl < 10 mm)	Flexion Instability^d^^*^ (nl < 1.0 mm)	Extension Instability^d^^*^ (nl < 1.0 mm)	C1–C2 Instability^e^ (nl < 4 mm)
		*Mean*						
Anxiety	190	2.68	72.07	2.60	41.39	4.51	4.61	7.47
Dizziness	179	2.70	69.30	2.69	41.31	4.46	4.34	7.46
Fatigue	205	2.71	71.41	2.58	40.88	4.41	4.44	7.41
Lightheadedness	178	2.68	71.71	2.77	40.89	4.44	4.38	7.44
Irritability	174	2.69	69.62	2.57	41.69	4.32	4.53	7.33
Insomnia	149	2.77	75.42	2.34	40.51	4.56	4.24	7.19
Neck grinding/cracking	194	2.70	70.07	2.53	40.69	4.55	4.27	7.48
Neck pain	215	2.72	69.91	2.56	40.96	4.41	4.32	7.37
Sleeping problems	179	2.74	75.11	2.52	40.71	4.53	4.38	7.23

IJV, internal jugular vein; CSA, cross-sectional area; nl, normal limit.

a Vagus nerve CSA was taken at C4–C5 level, as there was so much compression at the atlas (C1) that it could not be viewed with ultrasound.

b Depth of curve = horizontal distance in the sagittal plane from posterior inferior C4 vertebra to line drawn from posterior inferior C6 vertebra to top of dens (optimal is 7–17 mm).

c C6AI = horizontal distance in the sagittal plane of the posterior inferior C6 vertebra to anterior atlas (optimal is <10 mm).

d Normally, there is little (<1 mm) anterolisthesis or retrolisthesis with flexion and extension, but what constitutes “excessive” pathological movement is dependent on several variables, including symptomatology.

* Ref. Alvarez et al. (107), Copyright © 2022, The Author(s). Published by Wolters Kluwer Health, Inc. http://creativecommons.org/licenses/by-nc-nd/4.0/.

e C1–C2 facet joint = while some overhang of the C1–C2 facet joint on open mouth view is considered acceptable, people can be symptomatic even when the overhang is <2 mm on each side.

## Heart rate variability testing

Another beneficial measurement to obtain is heart rate variability (HRV), one of the best predictors of current health status, morbidity, mortality, and a risk factor for future illness, especially regarding cancer, heart disease, and sudden cardiac death (65). Vagus nerve activity can be observed in a noninvasive manner via the measurement of variability of interbeat cardiac intervals, called HRV. There are rings, finger probes, and watches available that can continuously monitor heart rate variability. While HRV measures the heart rate responses around the mean heart rate (generally from an electrocardiogram), in clinical practice, moment-to-moment fluctuations in pulse rate called “pulse rate variability” are utilized by various photoplethysmography sensors on wearable watch devices or finger probes (66). In the office or at home, the patient’s heart rate variability can be assessed during different head and neck motions, or even with different computer heights. HRV is strongly correlated with actual vagal nerve activity and how a person handles stress (67, 68). HRV abnormalities are seen in disorders from headache and schizophrenia to cancer, and its association with threat processing, emotional regulation, and executive functioning makes VN function vital to basically every bodily system and disease (69–72).

**Figure 10:**
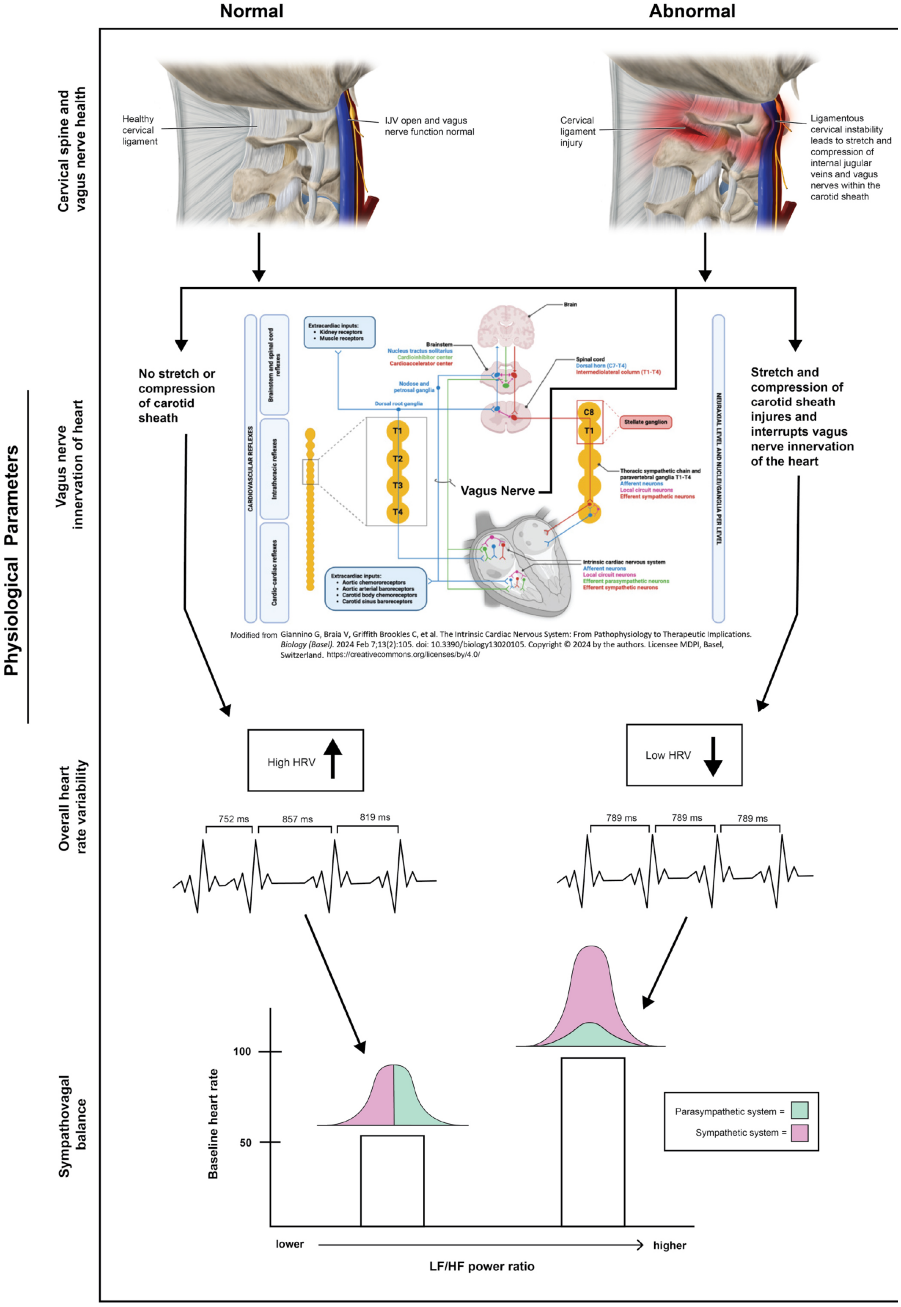
Connection between ligamentous cervical instability, the vagus nerve, and heart rate variability parameters.

HRV is an easy and inexpensive way to assess autonomic nervous system dysfunction. It measures the variability between “RR intervals,” or the time that elapses between 2 consecutive R-waves on an electrocardiogram. Spectral analysis of the RR interval provides a means of quantitating the variability of regular oscillations of the pulse interval over a range of frequencies. The spectral power (variability) is distributed within 3 major frequency bands: very low frequency (approx. 0.04 Hz in humans), low frequency (approx. 0.1 Hz), and high frequency (>0.15 Hz). Overall, sympathetic activity better correlates with the low frequency range (0.04–0.15 Hz), while parasympathetic activity is associated with the higher frequency range (0.15–0.4 Hz) of modulation frequencies of the heart rate. The ratio of low frequency to high frequency (LF/HF) is termed “sympathovagal balance.” Sympathetic dominance is signified by a higher LF/HF ratio, lower HRV, and higher resting heart rate, while a high vagal tone—or parasympathetic dominance—is typically seen by a lower LF/HF ratio, higher HRV, and lower resting heart rate (see Figure 10). HRV is very sensitive to a person’s breathing rate; generally, the slower the breathing rate, the higher one’s HRV (73, 74). HRV improvements can then be verified by therapies that help optimize cervical curve lordosis and stability, including specific chiropractic adjustments, physiotherapy, workstation ergonomics, and prolotherapy (75–80).

## Signs and symptoms of cervicovagopathic dysautonomia

Cervicovagopathic dysautonomia denotes dysautonomia due to vagus nerve pathology from a structural neck issue, but dysautonomia can have many causes. Autonomic dysfunction, or dysautonomia, is an improper functioning of the nerves of the autonomic nervous system. While this paper and study emphasize dysautonomia from parasympathetic hypoactivity from VN degeneration, sympathetic hypoactivity and/or parasympathetic hyperactivity may also be seen in dysautonomia or autonomic dysfunction (81). Dysautonomia can be primary, secondary, or idiopathic; secondary causes include hormone issues (diabetes), infections, autoimmunity, chronic diseases or pain, vascular origins, cardiac disease, or neurological conditions such as Alzheimer’s or Parkinson’s disease, as well as systemic structural conditions such as hypermobility disorders, including Ehlers-Danlos syndrome (82–84).

Dysregulation of the autonomic nervous system has the potential to affect the functioning of every organ of the body, including essential integrative systems such as arterial blood pressure, heart, digestion, and immune function, and body temperature. Imbalance of sympathetic to parasympathetic tone can lead to symptoms. Neurovascular dysautonomia, or hemodynamic instability of vascular origin, which is frequently seen in patients with joint hypermobility, causes autonomic dysfunction with sympathetic hyperactivity (85, 86). This neurovascular dysautonomia can occur from both an arterial component or one involving the cerebral or cervical venous system (84, 87, 88).

While the VN is the main component of the parasympathetic nervous system and makes up about 80–90% of the nerve fibers in the system, the many sympathetic system ganglia run alongside the anterior vertebral bodies from the upper neck to the coccyx. The face and head are specifically provided with sympathetic efferent innervation by the superior cervical sympathetic ganglion, which sits approximately at the level of the second and third cervical vertebrae (C2 and C3). While structural neck postures and disorders can impair the VN function, dysfunctional neck issues also potentially negatively affect the sympathetic ganglia and fibers in the cervical spine, especially the superior cervical sympathetic ganglia: another potential etiology for dysautonomia and chronic symptoms (2, 89, 90).

Dysautonomia is characterized by dysregulation of the autonomic nervous system, with a common pattern being sympathetic dominance or hypofunctioning of the parasympathetic nervous system or low vagal tone. While dysautonomia can contribute to distressing symptomatology in animal studies, VN degeneration can be so serious that widespread arterial vasospasm happens in the body, including the brain, lungs, heart, lymph nodes, and cervical nerves (91, 92). The amount of VN degeneration also correlates with animal survival. Everything that happens involuntarily in the body, including cardiovascular, gastrointestinal, genitourinary, ocular, respiratory, thermoregulatory, vasomotor, and homeostatic functions, and a host of other involuntary reflexes, can be affected by VN dysfunction and degeneration.

**Figure 11:**
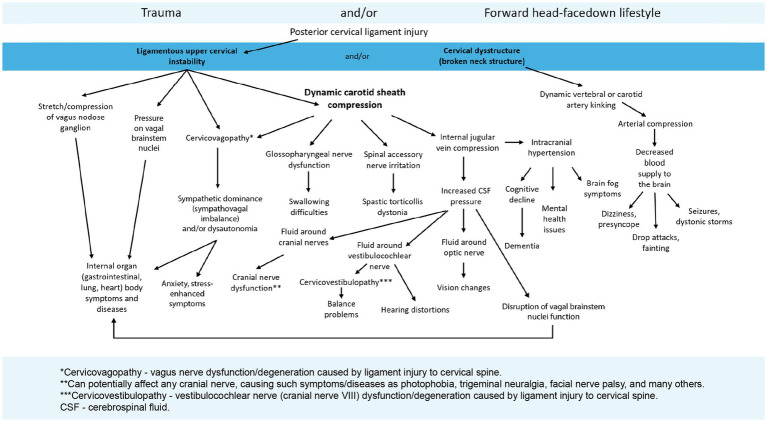
The potential symptoms from ligamentous cervical instability and the pathophysiology it causes, including vagus nerve degeneration and dysfunction.

The hallmark feature of many cases of dysautonomia may be dysfunction of the VN. This dysfunction can occur even without mechanical pressure on the VN but by the presence of stressors, including chronic pain, which suppress vagus nerve activity (83, 93, 94). The most common symptoms of VN dysfunction (cervicovagopathy and dysautonomia) include chronic pain, fatigue, dizziness, lightheadedness, a spinning or pulling sensation (in a particular direction), weight loss, poor focusing, exercise intolerance, emotional lability, inflammation, heartburn, bloating, diarrhea, tinnitus, headache, anxiety, depression, brain fog, swallowing difficulty, vision changes, and inability to handle stress well. Progressive compression of the carotid sheath by ligamentous cervical instability may be at the forefront of VN degeneration and the symptoms it causes (see Figure 11). This connection would also explain the association between increased cerebral venous pressure, intracranial pressure, and dysautonomia (87, 95).

One clue that a cervical cause of dysautonomia exists is cracking, popping, or grinding in the neck with motion. Muscle tightness and the feeling that the head is too heavy for the neck to support can also indicate instability. Symptoms can also increase when turning the head or when facial movements such as laughing, chewing, or speaking cause what we term “episymptoms,” which are symptoms that are manifested by activities that do not normally cause those symptoms. Episymptoms can include flushing, sweating, temperature dysregulation, headaches, vision changes, electric shocks, palpitations, tachycardia, or other autonomic symptoms. Signs include changes in blood pressure, impaired thermoregulation, fatigue, changes in mental state (such as an increase in stress or lightheadedness), uvula deviation to one side, an inability of the palate to raise normally, decreased gag reflex, and dilated pupils (96–98). Simple signs of VN dysfunction are a reduced or absent gag reflex, a deviated uvula, or a palate that does not elevate (or reduce) to say, “Ahh” (99). The sensory branch of the gag reflex is the glossopharyngeal nerve, and the motor branch is the vagus nerve. The VN innervates the main muscle that raises the soft palate, the levator veli palatini muscle, so if there is VN degeneration on one side, the palate is often higher on that side and the uvula deviates to the opposite side, but if the VN degeneration is bilateral, the palate exhibits decreased elevation to say, “Ahh,” and palate heights are even bilaterally.

## Improving vagus nerve function by dynamic structural medicine principles

**Figure 12:**
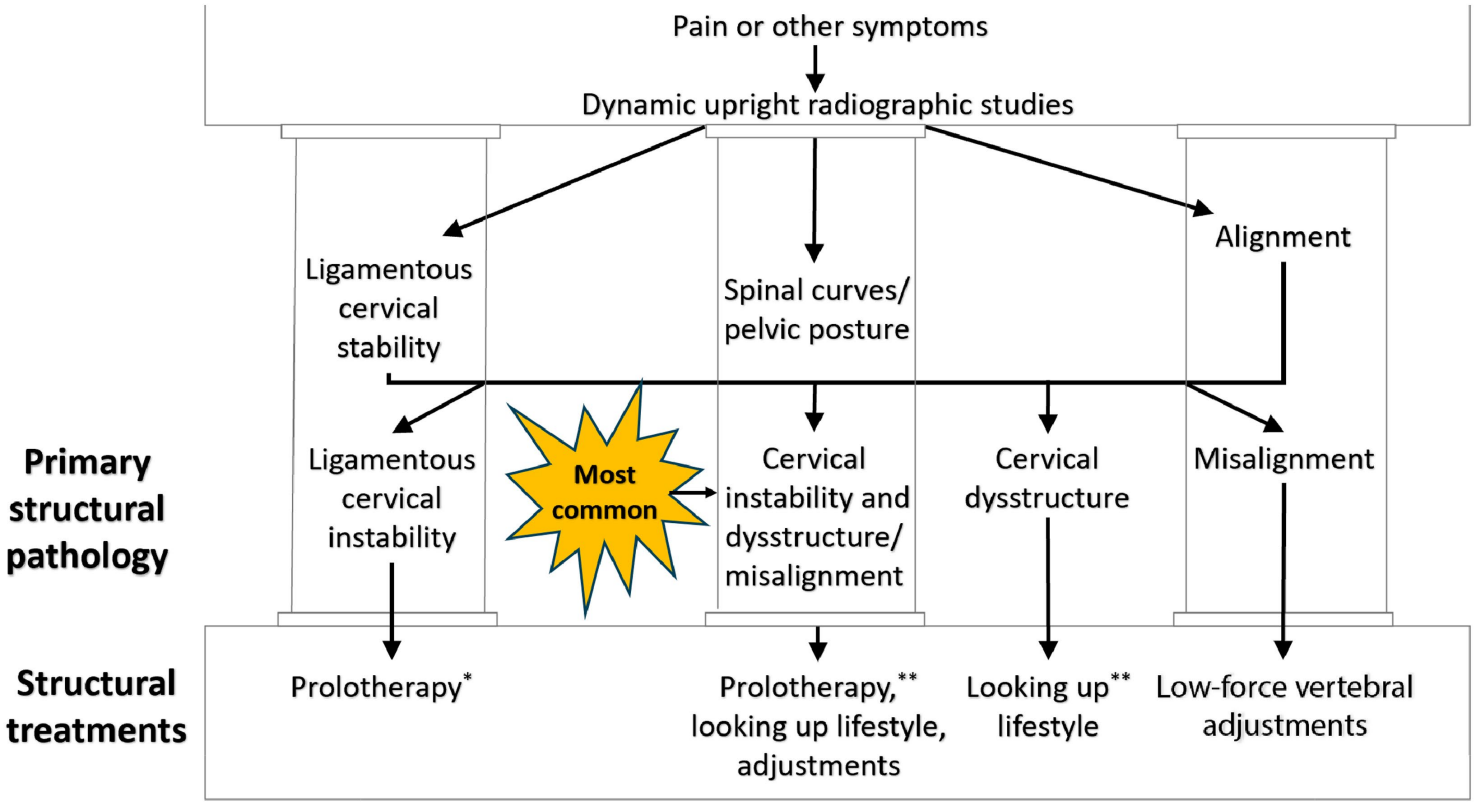
Cervical treatment recommendations based on dynamic upright radiographic studies. Patients often have a combination of ligamentous cervical instability, cervical dysstructure (breakdown of cervical curve), and misalignments, the 3 pillars of cervical structural health. * Some extreme cases of instability require surgery or other methods. ** Optimizing cervical curve is multifaceted and can include ergonomics, exercise, physical therapy, low-force adjustments, and many other physical medicine techniques.

**Figure 13:**
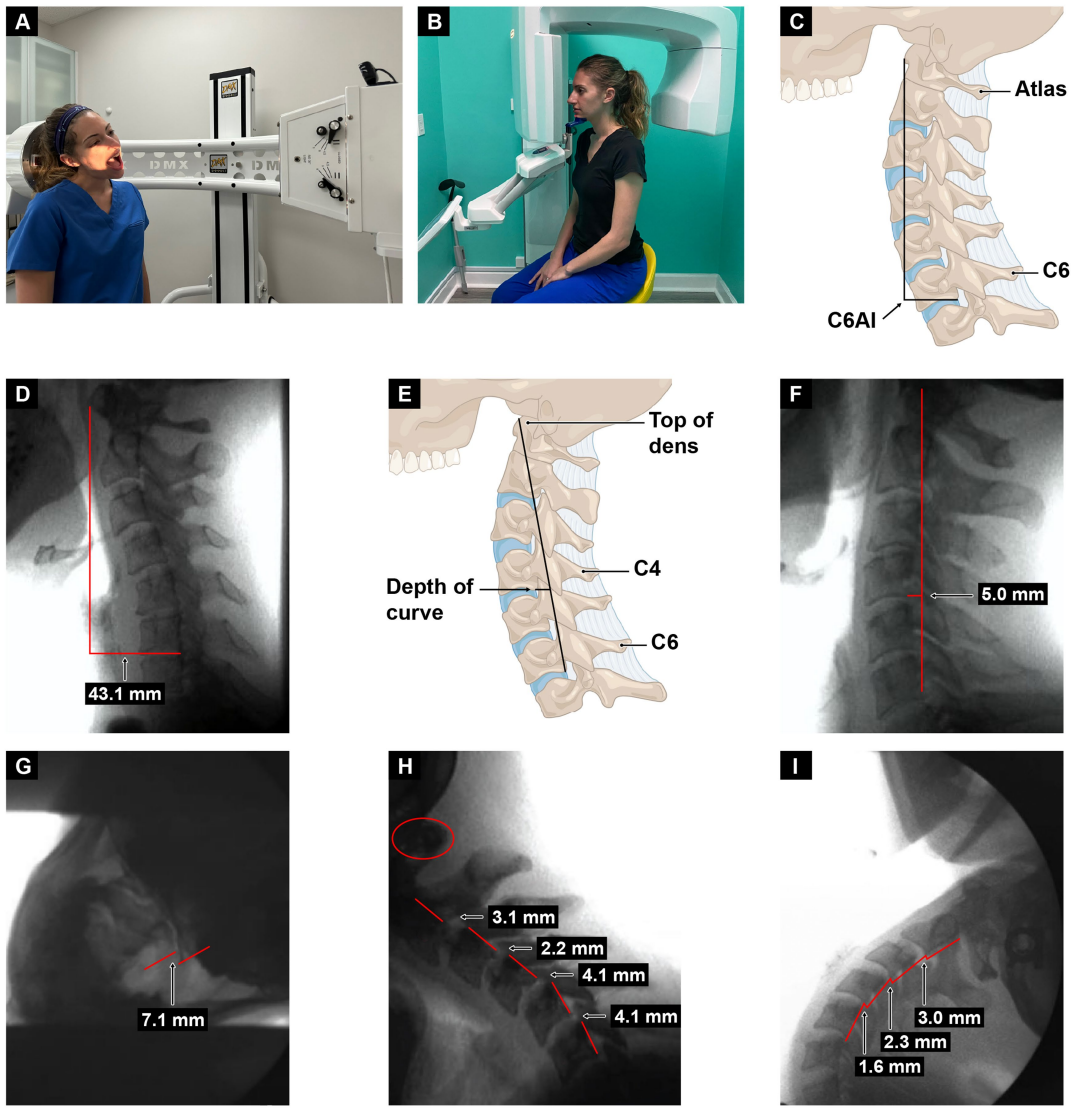
Upright digital motion (fluoroscopic) X-ray (DMX) and cone beam CT (CBCT) scan with structural measurements. **(A)** DMX positioning for open mouth lateral flexion. **(B)** CBCT setup. **(C)** Forward head (C6AI*) illustration. **(D)** C6AI measurement. **(E)** Depth of curve** illustration. **(F)** Depth of curve using DMX. **(G)** C1–C2 instability. **(H)** Flexion, lower cervical instability. **(I)** Extension, lower cervical instability. * C6AI = horizontal distance in the sagittal plane of the posterior inferior C6 vertebra to anterior atlas (optimal is <10 mm). ** Depth of curve = horizontal distance in the sagittal plane from posterior inferior C4 vertebra to line drawn from posterior inferior C6 vertebra to top of dens (optimal is 7–17 mm).

Dynamic structural medicine explains how human structure changes with different postures and motions to give the body health or disease. It involves looking at the 3 pillars of structural health: alignment, posture (spinal curves), and joint stability. Upright cervical motion x-ray (videofluoroscopy) and cone beam CT scanning can be used to determine these parameters for the cervical spine (see Figure 12). Depending on what is found, specific potentially corrective therapies can be prescribed. The cervical misalignment, dysstructure, and/or ligamentous instability can be treated by low-force adjustments (especially of the atlas), therapeutic exercises and ergonomics, and prolotherapy, respectively (see Figure 13). The hallmark of treatment is improvement of ergonomics related to computer and cell phone usage. Improvements in the cervical curve and stability can then be serially monitored.

Like a hinge holding a cabinet door, when one screw of the hinge loosens, every time the cabinet door is opened, not only is that first screw loosening further, but the other screws sustain increased force and are thus more prone to loosening. The best solution in this scenario is a screwdriver to tighten the screws. In the human body, ligaments act like the screws that stabilize a hinge (joint). Just like the progressive loose screw example, ligamentous cervical instability is a progressive disorder, as damage to one cervical ligament puts additional force on the adjacent ligaments. To stop ligamentous cervical instability from progressing, the best treatment is prolotherapy, which is like a screwdriver to tighten loose ligaments. Prolotherapy specifically targets the posterior ligament complex of the neck to induce a tightening and strengthening of the ligaments (100–102). Treatments are generally given every 4–6 weeks. For people with severe upper cervical instability, a period of cervical bracing may be necessary. The number of treatments depends on many factors, but in general, 4–10 sessions are necessary for cases of ligamentous cervical instability-caused cervicovagopathy. An improvement in the person’s structural parameters and neck vital testing, including VN cross-sectional diameters and HRV parameters, generally coincides with their clinical improvement. Cervical vertebral alignment, overall cervical curve, and ligamentous stability are all interrelated. In summary, *structural* treatments are needed for *structural* deficiencies, which may include targeting ligamentous cervical instability with prolotherapy and cervical curve correction for patients whose symptoms and/or diseases are related to VN degeneration.

## Case example

**Figure 14:**
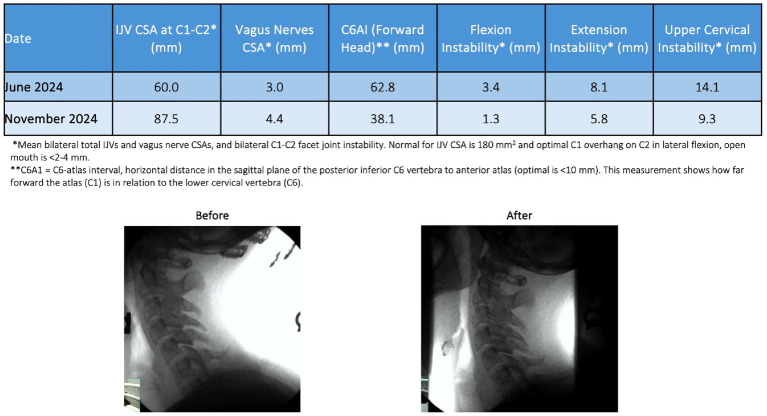
Improvement in a patient’s cervical curve structure and stability correlates with the opening of her jugular veins (IJVs) and improvement in cross-sectional areas (CSAs) of the vagus nerves. Over the course of treatment, many of her brain, body, and neck symptoms improved significantly.

A 27-year-old female presented to Caring Medical in June 2024 with a constellation of symptoms that occurred with increasing frequency over the course of the last 6 years, including disabling brain fog, anxiety, blurry vision, heart palpitations, headache, fatigue, digestive problems, panic attacks, sensitivity to sound, irritability, tinnitus, dizziness, and sleeping problems. Initial and follow-up cervical structural and neck vitals analyses are presented in tabular form (see Figure 14). Based on the findings of forward head posture, cervical curve correction protocol was initiated that included education on improved ergonomics of her workstation, laying on a Denneroll^®^, and various neck exercises and Prolotherapy for her multilevel ligamentous cervical instability. Over the course of 6 months, her cervical lordotic curve and stability improved. As of January 2025, she reported significant improvement in all presenting symptoms, specifically 80–95% improvement in vertigo, brain fog, anxiety, digestive problems, heart palpitations, and panic attacks, and 50–75% improvement in fatigue, tinnitus, sound sensitivity, and irritability. This case example showed a positive correlation between the treatment of cervical spine dysfunction, improvement in her vagus nerve cross-sectional area, and a decrease of many of her symptoms.

## Discussion

Vagal nerve dysfunction has been implicated in the etiology of many symptoms and diseases, including dysautonomia. The pathology of VN dysfunction that has its etiology in the neck is termed cervicovagopathy. VN degeneration can be documented by measuring VN cross-sectional area by carotid sheath ultrasound, and autonomic nervous system dysfunction by HRV testing. Low HRV and a high LF/HF ratio parameter are seen as confirming an imbalance in the sympathetic/parasympathetic equilibrium. The forward head-facedown lifestyle (especially due to excessive computer and cell phone usage) stretches the posterior ligament complex of the cervical spine, which can ultimately lead to a breakdown of the cervical lordotic curve (dysstructure), which puts excessive stretch and compression on the VN, leading to dysfunction or degeneration. Disorders and diseases with a VN component may necessitate corrective cervical *structural* therapies. Dynamic structural medicine principles note that atlas misalignments, cervical dysstructure, and ligamentous cervical instability—especially at the atlanto-axis (C1–C2)—need to be resolved to decrease the destructive forces on the VN.

## Clinical relevance and future directions

Our findings provide new insights into the potential structural causes of vagus nerve dysfunction and degeneration, and thus *chronic* autonomic dysfunction. Traditional approaches for conditions of autonomic dysfunction, including postural orthostatic tachycardia syndrome and dysautonomia, emphasize pharmacological and non-pharmacologic treatments that reduce symptoms, but the actual underlying etiology often remains elusive (103, 104). As vagus nerve cross-sectional areas are easily measured by B-mode ultrasound, future research should investigate their correlation with standard testing for autonomic dysfunction, including heart rate variability testing, tilt table tests, quantitative sudomotor axon reflex testing, and Valsalva maneuvers. The improvement in *chronic* symptoms and VN cross-sectional areas in patients with autonomic dysfunction that occurred after cervical curve correction programs could validate the connection between the cervical lordotic curve, vagus nerves, and a balanced, healthy autonomic system. This confirmation would open up a new avenue of objective testing in those patients with autonomic and cervical structural dysfunction and treatment regimen with potential long-term solutions, which could be expedited by the clinical integration of artificial intelligence to enhance the efficiency and speed at which healthcare problems associated with dysfunctions of the vagus nerve and cervical spine are appropriately diagnosed and addressed (105, 106).

## Data Availability

The datasets presented in this article are not readily available because of ethical and privacy restrictions. Requests to access the datasets should be directed to the corresponding author.
